# Benchmarking open source and paid services for speech to text: an analysis of quality and input variety

**DOI:** 10.3389/fdata.2023.1210559

**Published:** 2023-09-20

**Authors:** Antonino Ferraro, Antonio Galli, Valerio La Gatta, Marco Postiglione

**Affiliations:** Department of Electrical Engineering and Information Technology, University of Naples “Federico II”, Naples, Italy

**Keywords:** ASR, speech to text, speech recognition, benchmark, multimedia

## Abstract

**Introduction:**

Speech to text (STT) technology has seen increased usage in recent years for automating transcription of spoken language. To choose the most suitable tool for a given task, it is essential to evaluate the performance and quality of both open source and paid STT services.

**Methods:**

In this paper, we conduct a benchmarking study of open source and paid STT services, with a specific focus on assessing their performance concerning the variety of input text. We utilizes ix datasets obtained from diverse sources, including interviews, lectures, and speeches, as input for the STT tools. The evaluation of the instruments employs the Word Error Rate (WER), a standard metric for STT evaluation.

**Results:**

Our analysis of the results demonstrates significant variations in the performance of the STT tools based on the input text. Certain tools exhibit superior performance on specific types of audio samples compared to others. Our study provides insights into STT tool performance when handling substantial data volumes, as well as the challenges and opportunities posed by the multimedia nature of the data.

**Discussion:**

Although paid services generally demonstrate better accuracy and speed compared to open source alternatives, their performance remains dependent on the input text. The study highlights the need for considering specific requirements and characteristics of the audio samples when selecting an appropriate STT tool.

## 1. Introduction

Automatic Speech Recognition (ASR) systems have become increasingly popular and are widely used in various applications such as virtual assistants, automated call centers, and speech-to-text transcription (Malik et al., 2021). The performance of ASR systems is heavily dependent on the quality and quantity of the data used for training and evaluation (Haeb-Umbach et al., 2020).

In this paper, we evaluate the performance of several state-of-the-art ASR models on seven commonly used datasets:

- LibriSpeech (Panayotov et al., 2015)
- Common Voice (Ardila et al., 2020)
- TED-LIUM (Rousseau et al., 2012)
- TIMIT (Zue et al., 1990)
- CHiME-5 (Barker et al., 2018)
- and WSJ0 (Drude et al., 2019).

The ASR models evaluated in this study include several open-source tools such as *Conformer* (Gulati et al., 2020), *HuBERT* (Hsu et al., 2021), *SpeechBrain* (Ravanelli et al., 2021), *WhisperX* (Bain et al., 2023), and *SpeechStew* (Chan et al., 2021), as well as paid tools such as *Amazon Transcribe*,^1^ *Azure Speech-to-Text*,^2^ *Google Speech-to-Text*,^3^ and *IBM Watson Speech to Text*.^4^

The evaluation metric used for comparing the performance of the ASR models is the Word Error Rate (WER), which is a widely used metric for evaluating ASR systems (Hamed et al., 2023). The main objective of this study is to provide a comprehensive evaluation of state-of-the-art ASR models on various datasets and to identify the best performing models for each dataset. The results of this study will provide valuable insights into the performance of ASR models and help researchers and practitioners in choosing the best ASR model for their specific application.

The paper's structure is as follows: Section 2 covers Speech-to-Text Systems, which is categorized into Open Source and Paid Services. Section 3 provides a comprehensive description of the utilized datasets. Section 4 presents the evaluation metrics employed for assessing the models. The Discussion of results is presented in Section 5, and Section 6 is dedicated to the Conclusions and Future works.

## 2. Speech-to-text systems

### 2.1. Open source

#### 2.1.1. DeepSpeech

Mozilla DeepSpeech (Hannun et al., 2014) is an open-source speech recognition platform that leverages deep learning technology to provide human-like accuracy in transcribing and converting audio files into text. The technology utilizes a powerful neural network model, trained on a vast amount of data, to achieve high levels of accuracy in transcribing speech. One of the key strengths of Mozilla DeepSpeech is its ability to learn and adapt to new languages and dialects.

Moreover, Mozilla DeepSpeech prioritizes privacy and security, and ensures that user data remains encrypted and secure at all times. This is an important feature, particularly for businesses and individuals working with sensitive or confidential information.

#### 2.1.2. Conformer: convolution-augmented transformer for speech recognition

Convolution neural networks and transformers are used in the Conformer model (Gulati et al., 2020) to generate a system for speech recognition that is more effective and efficient. Its ability to handle lengthy audio input sequences, use of multi-head self-attention mechanisms to capture complex relationships between various parts of the input, and incorporation of convolutional layers to capture local patterns in the input are some of its key characteristics.

The Conformer model additionally employs dropout regularization to lessen overfitting and boost generalization efficiency.

#### 2.1.3. HuBERT: self-supervised speech representation learning by masked prediction of hidden units

HuBERT (Hsu et al., 2021) is a self-supervised method for learning speech representations that makes use of offline clustering to produce aligned target labels for a prediction loss that is similar to BERT. In order to forecast specified cluster assignments, the model uses masked continuous speech characteristics, and the predictive loss is only applied across the masked regions. In order to accurately infer the targets of masked inputs, the model is compelled to acquire good high-level representations of unmasked inputs.

HuBERT's primary advantage is its ability to solve three specific problems in self-supervised speech representation learning: the presence of many sound units in each input utterance, the lack of a lexicon of input sound units during pre-training, and the variable length and lack of explicit segmentation of sound units.

#### 2.1.4. SpeechBrain

SpeechBrain (Ravanelli et al., 2021) is an open-source tool that offers a range of features for speech processing beyond the basic functionalities. These include multi-GPU training, which supports both DataParallel and DistributedDataParallel modules, allowing the use of GPUs on the same and different machines. Automatic mixed-precision can be enabled by setting a single flag to reduce the memory footprint of the models.

Additionally, SpeechBrain supports PyTorch's Just-In-Time (JIT) compiler for native compilation. The toolkit also extends WebDataset with on-the-fly dynamic batching and bucketing, enabling efficient batching in sequential shard-based data reading, which is necessary for processing large corpora on network filesystems. Specifically, SpeechBrain includes implementations of several popular automatic speech recognition (ASR) models, including hybrid models that combine deep neural networks with Hidden Markov Models (HMMs), as well as end-to-end models that directly map audio to text using neural networks (e.g., wav2vec 2.0; Baevski et al., 2020).

SpeechBrain also provides pre-trained models for speech recognition, which can be fine-tuned or adapted to specific domains or languages. With these modules, SpeechBrain can perform high-quality speech-to-text transcription for a wide range of applications, such as dictation, voice search, and voice-controlled interfaces.

#### 2.1.5. WhisperX

WhisperX (Bain et al., 2023) is a time-accurate speech recognition system with word-level timestamps that utilizes voice activity detection and forced phoneme alignment. It is designed to efficiently transcribe long-form audio with accurate word-level timestamps. In particular, WhisperX segments the input audio for transcription by first pre-segmenting it with an external Voice Activity Detection (VAD) model.

The resulting VAD segments are then cut and merged into approximately 30-s input chunks with boundaries that lie on minimally active speech regions. This enables batched whisper transcription, which is transcribed in parallel with Whisper and forced aligned with a phone recognition model to produce accurate word-level timestamps at high throughput.

#### 2.1.6. SpeechStew

SpeechStew (Chan et al., 2021) is an English speech recognition model that combines various publicly available datasets to achieve state-of-the-art results without the use of an external language model. It is a simple approach to end-to-end speech recognition, which leverages both multi-domain training and transfer learning.

SpeechStew follows a simple recipe of combining all available speech recognition data without any domain-dependent re-balancing or re-weighting, and trains a single large neural network on the combined data. Additionally, SpeechStew learns powerful transfer learning representations, which allows it to fine-tune on new unseen tasks with strong empirical results. For more details, refer to Table 1.

**Table 1:** Related work, pros and cons.

**Paper**	**References**	**Year**	**Dataset**	**Pros**	**Cons**
DeepSpeech	Hannun et al. (2014)	2020	Switchboard and Fisher corpora	Specialized components not required for speaker adaptation	Performance in different environments
Conformer	Gulati et al. (2020)	2020	LibriSpeech	Performance with a small model (10M parameters)	Training and optimization of Conformer architecture
HuBERT	Hsu et al. (2021)	2021	Libri-light and LibriSpeech	Scalability and Transferability	The availability of unlabeled data may have an impact on performance
Speechbrain	Ravanelli et al. (2021)	2021	TIMIT, LibriSpeech and Common Voice	Competitive performance in different use cases	Potential privacy problems
WhisperX	Bain et al. (2023)	2023	AMI Meeting Corpus	Decreases hallucination and repetition in long audio transcriptions	Outstanding speech recognition performance in different domains and languages
SpeechStew	Chan et al. (2021)	2021	Mixed datasets (including commercial/private)	Excellent results without an external language model	Availability of computing resources

### 2.2. Paid services

#### 2.2.1. Amazon transcribe

Amazon Transcribe is a highly effective speech-to-text service offered by Amazon Web Services (AWS). It utilizes deep learning technology to enable automatic speech recognition (ASR) and transcription of audio recordings. The service has proven its effectiveness across various range of sources, including telephone conversations, lectures, interviews, and podcasts, among others. In addition, Amazon Transcribe supports a wide range of audio input formats, including MP4, WAV, and FLAC, among others. Additionally, since it is integrated in the AWS suite, it can output transcriptions in plain text but also directly on Amazon S3 or Amazon CloudWatch logs. This flexibility in audio input/output formats allows for easy integration with a variety of other services and applications.

Amazon Transcribe pricing is mostly based on the amount of audio data that is transcribed per month. The pricing model offers a pay-as-you-go approach with no upfront costs or minimum fees. In particular, there is a free tier for Transcribe, which allows users to transcribe up to 60 min of audio per month at no cost for the first 12 months of the account. Beyond that, as of September 2021, the cost of Amazon Transcribe is 0.024$min for the first 250,000 min.

#### 2.2.2. Microsoft Azure Speech to Text

Microsoft Azure Speech to Text is a powerful cloud-based service that uses advanced neural network models to transcribe spoken language into text. Besides transcription, the tool has additional capabilities, including subtitling, or translation. The service is multi-language and can even guarantee high accuracy in a variety of accents and low-resource languages. Additionally, Azure Speech to Text boasts low latency, providing near real-time transcription results. Interestingly, Azure Speech to Text also offers a variety of customizable features that can be tailored to individual needs. For example, users can adjust parameters based on the audio quality, the number of speakers. Also, users can also implement personalized acoustic and language models, improving transcription accuracy and reducing errors related to spoken language data that is specific to a particular industry or domain.

Finally, as with other cloud-based services, the cost of using Azure Speech to Text is based on usage, differentiated by processing minutes, i.e., billed per minute of audio transcribed. It also offers pay-as-you-go pricing with no upfront costs or long-term commitments, making it an affordable choice for users of all sizes. In addition, there is also a free tier including five audio hours per month.

#### 2.2.3. Google Cloud Speech API

The Google Cloud Speech API is a powerful tool for converting audio data into digital text. The API is supported by Google's advanced deep neural network models, which enable it to achieve excellent accuracy rates even in challenging audio environments (e.g, noisy environments, audio distortions). The API can handle a variety of audio inputs, including live audio streams and pre-recorded audio files, and is capable of recognizing and transcribing speech in over 120 languages and dialects.

In addition to its powerful speech recognition capabilities, the Google Cloud Speech API is also highly scalable, allowing it to handle large volumes of audio data in real-time. This scalability makes it suitable for a broad range of applications, including voice-activated virtual assistants, speech-to-text transcription for video or audio content, and real-time captioning for live events.

The service offers a free tier similar to AWS Transcript, i.e., 60 free minutes per month. After that, the pricing strategy depends on the processing minutes, i.e., 0.024$min. Interestingly, the service explicitly advertises a medical tier for healthcare applications at the same cost of the standard one.

#### 2.2.4. IBM Watson Speech to Text

IBM Watson Speech to Text is a cutting-edge technology that enables businesses and individuals to accurately transcribe and convert audio and video files into text. This technology utilizes advanced machine learning algorithms to ensure high accuracy and reliability, even for large or complex audio files.

One of the key strengths of IBM Watson Speech to Text is its ability to understand natural language and dialects from a variety of sources, including phone calls, videos, and live conversations. This allows businesses to gain valuable insights from their customer interactions, such as analyzing sentiment and identifying key trends or behaviors. This technology can transcribe over 100 languages and dialects, making it an ideal solution for international organizations.

In addition to its power and flexibility, IBM Watson Speech to Text is also highly scalable and can be easily integrated into existing workflows and systems. The technology is designed to be user-friendly, with intuitive interfaces and robust documentation and support to ensure that businesses can quickly and easily get up and running.

## 3. Datasets

Speech recognition is one of the most fascinating and challenging fields of research in artificial intelligence. It aims to develop algorithms that enable computers to accurately recognize and transcribe spoken language. The applications of speech recognition are numerous, including virtual personal assistants, voice-operated interfaces, automatic captioning for video content, and voice-authentication systems.

To develop effective speech recognition models, large and diverse datasets are required. These datasets serve as the foundation for training and testing machine learning algorithms. In recent years, several publicly available datasets have been released to support speech recognition research. Some of the most well-known datasets include the TIMIT corpus, the Common Voice dataset, the VoxCeleb dataset, and the LibriSpeech dataset. These datasets contain thousands of hours of audio recordings from a variety of speakers, accents, and languages, making them valuable resources for researchers and developers in the field.

By leveraging these datasets and powerful machine learning techniques, researchers are making rapid progress in speech recognition, bringing us closer to the goal of developing sophisticated and reliable speech recognition systems that can understand and transcribe human language with exceptional accuracy. The following sections describe the most famous dataset used for the task under analysis.

### 3.1. LibriSpeech

To aid in automated speech recognition (ASR) research, the LibriSpeech corpus (Panayotov et al., 2015) is a collection of publicly accessible audiobooks that have been transcribed and segmented. Over 1,000 h of speech from 2,484 different speakers are included in the corpus, which covers a wide range of topics from numerous literary genres.

The corpus is intended to reflect the variety and complexity of real-world speech and to be indicative of genuinely spoken language. The recordings were from a variety of audiobooks, including fiction, non-fiction, and technical volumes, and they featured a variety of speakers with a range of ages, genders, and accents. The recordings were transcribed using a forced-alignment process that ensured that each word in the speech signal was correctly segmented and labeled with its corresponding text.

The transcriptions are provided in plain text format, with accompanying metadata that includes information about the speaker, book, and chapter. The corpus has been divided into multiple sets of development and test data to facilitate ASR research, with different subsets meant to imitate various real-world situations like loud settings and speaker variability. A set of sentences that are phonetically balanced and part of the corpus can be utilized to train and test ASR systems.

### 3.2. Common Voice Dataset

The Common Voice Dataset (Ardila et al., 2020), created by Mozilla, is a publicly accessible compilation of human voices intended for use in training machine learning models for speech-to-text transcription. This dataset is a valuable asset for individuals engaged in the development and exploration of speech recognition technology, presenting a plethora of voices recorded across varied accents and languages.

The Common Voice Dataset's diversity renders it a useful means of ameliorating the precision of speech recognition algorithms through aiding researchers and developers. However, the set presents difficulties such as the presence of pronounced foreign words, pauses, noise, reverberation, and various recording artifacts.

The Common Voice project employs crowd-sourcing for both data collection and data validation. Community members use the provided tools to translate the interface, submit text sentences, and finally record and validate voices in their new language. The recordings were made in a variety of environments, including homes, offices, and public spaces. The validation process involved multiple rounds of listening to recordings by different community members to ensure quality control. The overall approach allows for organic scaling to new languages as more community members participate in the project. In fact, originally encompassing 29 languages, the Common Voice Dataset has grown to include a total of 38 languages, as of November 2019, with over 50,000 individuals participating, that has led to the acquisition of 2,500 h of audio content. The dataset is continually advancing, with the latest English release, Common Voice 13.0, comprising 3,209 recorded hours and 86,942 voices.

### 3.3. TED-LIUM

The TED-LIUM Automatic Speech Recognition Corpus (Rousseau et al., 2012) is a corpus developed by the LIUM for Automatic Speech Recognition (ASR), based on the TED Talks. It was built during the IWSLT 2011 Evaluation Campaign and is composed of 118 h of speech with its accompanying automatically aligned transcripts. The data for the TED-LIUM Corpus was extracted from the freely available video talks on the TED website. The corpus was built in an unsupervised way, based on iterations refining the alignment between audio data and raw text from closed captions.

A manually transcribed development corpus accompanies the training corpus, for a total of 4 h of speech. Overall, the dataset contains non-native speech and challenging acoustic conditions due to the recordings being made in a variety of venues and with diverse recording equipment, which can make it difficult for automatic speech recognition models to accurately transcribe the talks. The dataset evolved overtime until its third release, TED-LIUM 3 (Hernandez et al., 2018), which offers more than double the amount of data for training acoustic models in English speech recognition compared to the previous releases of the corpus.

### 3.4. TIMIT

The TIMIT dataset (Zue et al., 1990) is a widely used speech corpus that was designed to facilitate research in the field of automatic speech recognition (ASR) and related areas. It consists of recordings of 630 speakers (430 males and 200 females) from eight major dialect regions of the United States, each reading ten carefully selected phonetically rich sentences. The recordings were made in a soundproof booth using high-quality microphones and a 16-bit digital recording system at a sampling rate of 16 kHz.

The TIMIT corpus includes both clean and noisy speech recordings, with noise artificially added to simulate realistic acoustic conditions. In addition to the speech recordings, the TIMIT dataset also provides a range of annotations and metadata, including phonetic transcriptions of the sentences, word-level transcriptions, speaker and dialect information, and detailed acoustic measurements such as formant frequencies and spectral features. These annotations enable researchers to perform a wide range of tasks, such as speaker identification, phoneme recognition, and language modeling, and to compare the performance of different algorithms and models on a standardized testbed.

Since its release in 1986, the TIMIT corpus has become a benchmark dataset in the field of ASR, and has been widely used in research and development of speech recognition systems. Its availability and standardization have also contributed to the reproducibility and comparability of results across different studies and research groups.

### 3.5. CHiME-5

The CHiME-5 dataset (Barker et al., 2018) is a mostly used speech corpus that was designed to address the problem of speech recognition in noisy and reverberant environments. It was created as part of the 5th CHiME (Computational Hearing in Multisource Environments) challenge, which aimed to promote research in the field of distant speech recognition. The CHiME-5 corpus consists of recordings of 16 speakers (8 males and 8 females) in a variety of real-world settings, such as homes, cafes, and offices. The speakers were recorded using a set of microphones placed at different positions and orientations, which were used to capture the speech signals as well as the surrounding noise and reverberation.

The dataset includes both clean and noisy speech recordings, with noise artificially added to simulate realistic acoustic conditions. In addition to the speech recordings, the CHiME-5 dataset also provides a range of annotations and metadata, including phonetic transcriptions of the sentences, speaker and channel information, and detailed acoustic measurements such as room impulse responses and noise statistics. These annotations enable researchers to perform a wide range of tasks, such as speaker identification, speech enhancement, and language modeling, and to compare the performance of different algorithms and models on a standardized testbed.

Since its release in 2018, the CHiME-5 corpus has become a valuable resource for researchers working on speech recognition in noisy and reverberant environments. Its availability and standardization have also contributed to the reproducibility and comparability of results across different studies and research groups.

### 3.6. WSJ0

The WSJ0 dataset (Drude et al., 2019) is a widely used speech-to-text dataset for acoustic modeling tasks in the field of Automatic Speech Recognition (ASR). It consists of approximately 80 h of speech training data from read speech by both male and female speakers, recorded in 1992. The dataset includes transcriptions of the spoken text in a standardized orthographic format, as well as supplementary information such as speaker IDs and gender labels.

The recordings are sampled at 8kHz with 16-bit resolution and are stored in WAV format. The WSJ0 dataset is divided into three subsets: training, development, and testing, with a total of 7138, 503, and 330 spoken utterances, respectively. The WSJ0 dataset has been widely used to train and evaluate various acoustic models for ASR, such as Hidden Markov Models (HMM) and Neural Network-based models. It has played a critical role in advancing research in the field of ASR, particularly in the development of deep learning-based approaches that have achieved state-of-the-art performance.

## 4. Evaluation

As most previous work (Hannun et al., 2014; Ravanelli et al., 2021; Bain et al., 2023), we consider the word error rate (WER) to evaluate the performance of speech-to-text models. In particular, the metric measures the percentage of words that are incorrectly transcribed by the model relative to the total number of words in the reference transcript. WER is calculated by summing the number of substitutions, deletions, and insertions made by the model and dividing that sum by the total number of words in the reference transcript. Formally,


$$\begin{array}{l} WER=\frac{S+D+I}{N}, \end{array}$$

where *S* is the number of substitutions, *D* is the number of deletions, *I* is the number of insertions, and *N* is the total number of words in the reference transcript.

Concretely, WER is a useful metric for comparing the performance of different speech-to-text models on a given dataset. It provides a quantitative measure of how well a model is able to transcribe speech and can help identify areas for improvement. However, it is important to note that WER alone does not provide a complete picture of a model's performance because of the following limitations:

- WER treats all errors equally. For example, a substitution of a common word for a less common word may be less significant than a substitution of a content word for a function word;
- WER does not account for semantic errors, i.e., errors that change the meaning of the transcript. For example, a model may correctly transcribe a sentence, but misunderstand the speaker's intent or misinterpret the context, resulting in a semantically incorrect transcript.

Overall, while WER has its limitations, it remains the most widely used and accepted metric for evaluating the performance of speech-to-text models. Its simplicity and standardization make it a convenient and efficient metric, and it provides a useful measure to compare the accuracy of open-source and paid STT services (Ali and Renals, 2018).

## 5. Results

In Table 2, we present the performance of open-source speech-to-text models. Our analysis reveals that the absolute word error rate (WER) performance of these models is significantly influenced by the dataset used for evaluation. Specifically, we find that the LibriSpeech dataset exhibits the lowest WER performance, likely due to its phonetically balanced recordings. Conversely, the CHiME-5 dataset poses a more challenging task for all models, owing to the high level of noise and reverberation present in the recordings.

**Table 2:** Word Error Rate (WER) achieved with open-source methods on the benchmarking datasets.

**Method**	**References**	**Variant**	**LibriSpeech (clean)**	**LibriSpeech (other)**	**CommonVoice**	**TED-LIUM**	**WSJ**	**CHiME-5**
DeepSpeech	Hannun et al. (2014)	—	7.3	21.5	43.8	18.9	6.5	75.3
Conformer	Gulati et al. (2020)	S	2.1	5.0	12.6	5.5	1.9	46.1
		M	2.0	4.3	12.1	5.2	1.8	45.2
		L	1.9	3.9	11.4	4.9	1.8	**38.1**
HuBERT	Hsu et al. (2021)	Large	1.9	3.3	11.5	4.9	1.4	44.3
		X-Large	1.8	**2.9**	10.9	**4.7**	1.4	42.8
SpeechBrain	Ravanelli et al. (2021)	CTC+Att	2.5	5.8	14.8	6.4	2.1	48.9
		CTC+Att+SSL	2.4	4.0	14.5	6.1	2.1	46.3
WhisperX	Bain et al. (2023)	Large-v2	3.1	8.9	21.1	9.7	2.9	62.7
SpeechStew	Chan et al. (2021)	Light	**1.7**	3.3	**10.8**	5.7	**1.3**	38.9
		Base	**1.7**	3.3	12.1	5.3	**1.3**	40.6

Methods are reported in chronological order. Best results are reported in bold.

Our results indicate that there is no single best model for all datasets, as each model exhibits optimal performance on a specific dataset. This observation highlights the critical role played by the dataset in determining model performance. Our analysis further identifies three main factors that impact performance: (i) dataset variability, which encompasses aspects such as audio quality, speaker accents, background noise, and domain-specific vocabulary; (ii) model architecture, with transformer-based models outperforming older sequential neural network models like RNNs and LSTMs; and (iii) training data, where the domain remains a critical factor that influences the generalization power of the models.

Interestingly, we observe that the size of transformer-based models does not significantly affect their WER performance. For example, SpeechStew *light* and *base* models demonstrate similar performance on both the LibriSpeech and WSJ datasets. Similarly, the Conformer *small* (S), *medium* (M), and *large* (L) models achieve comparable performance on both the LibriSpeech and TEDLIUM datasets.

In Table 3, we present the results of comparing various paid speech-to-text services, including Amazon Transcribe, Microsoft Azure, Google Cloud Speech, and IBM Watson. Our findings suggest that Amazon Transcribe and Microsoft Azure are the better choices in terms of performance. Notably, when looking at the cost for limited usage (less than 2,000 h per month) Microsoft Azure is also the cheaper option, costing only 0.013 $/min compared to Amazon Transcribe's 0.024 $/min.

**Table 3:** Word Error Rate (WER) achieved with paid services on the benchmarking datasets.

**Method**	**Price (USD/min)**	**LibriSpeech (clean)**	**LibriSpeech (other)**	**CommonVoice**	**TEDLIUM**	**WSJ**	**CHiME-5**
Amazon Transcribe	0.024	5.2	**9.6**	15.9	**4.3**	**1.4**	**40.3**
Microsoft Azure	0.013	**5.0**	9.7	**12.1**	5.0	1.6	40.6
Google Cloud Speech API	0.024	6.6	13.6	18.4	6.7	2.9	49.1
IBM Watson Speech to Text	0.02	11.1	26.4	38.3	11.9	4.3	61.2

Best results are reported in bold.

However, our analysis also highlights that open-source solutions outperform paid services for most datasets, including LibriSpeech, CommonVoice, WSJ, and CHiME. This probably depends on the fact that open-source solutions can be customized and adapted to specific use cases, while paid services are typically optimized for general-purpose use cases. Additionally, open-source solutions, especially those based on modern neural network models (e.g., SpeechStew, HuBERT), often adopt *ad hoc* optimized training techniques, resulting in better performance on benchmark datasets.

One of the primary constraints of current speech-to-text models is their reliance on significant amounts of labeled data, which can be both time-consuming and costly to acquire. To address this limitation, future research could concentrate on investigating novel techniques that can reduce the amount of labeled data required for training, such as unsupervised or self-supervised learning approaches. Furthermore, the deployment of speech-to-text models in real-world scenarios raises several challenges related to privacy, security, and ethical considerations. Future research could explore these challenges and develop solutions to ensure the responsible and ethical use of speech-to-text technology.

It is important to note that the choice between paid and open-source services is not solely based on performance. Other factors, such as usability, support, documentation, and access to proprietary datasets and models, may also influence the decision. For example, paid services may have better support and be easier to integrate with other services, and may perform better in niche domains such as healthcare or surveillance. Ultimately, the choice between paid and open-source solutions depends on the specific needs and constraints of the user.

## 6. Conclusions and future works

In this study, we compared the performance of open-source and paid speech to text models across various datasets. Our results show that the performance of these models is largely dependent on the characteristics of the dataset, and that each model performs best on a different dataset. We also found that the use of modern transformer-based architectures outperforms older models based on sequential neural networks, and that the choice between paid and open-source services depends on various factors such as performance, usability, and availability of proprietary datasets.

Despite the progress made in recent years, there is still room for improvement in speech to text models. One of the main limitations of current models is their dependence on large amounts of labeled data, which can be time-consuming and expensive to obtain. Future research could focus on exploring new techniques to reduce the amount of labeled data needed for training, such as unsupervised or self-supervised learning approaches.

Another area for future research is the development of speech to text models that are more robust to variations in the input signal, such as different speaker accents, background noise, or domain-specific vocabulary. This could involve the use of transfer learning or domain adaptation techniques, as well as the creation of more diverse and representative datasets for model training.

Finally, the deployment of speech to text models in real-world scenarios raises several challenges related to privacy, security, and ethical considerations. Future research could explore these challenges and develop solutions to ensure the responsible and ethical use of speech to text technology.

## Data availability statement

Publicly available datasets were analyzed in this study. Details are included in the citations/references.

## Author contributions

AF, AG, VL, and MP: conception and design of study, acquisition of data, analysis and interpretation of data, drafting the manuscript, revising the manuscript critically for important intellectual content, and approval of the version of the manuscript to be published.

## Footnotes

^1^ https://aws.amazon.com/transcribe/

^2^ https://azure.microsoft.com/en-us/products/cognitive-services/speech-to-text/

^3^ https://cloud.google.com/speech-to-text

^4^ https://www.ibm.com/cloud/watson-speech-to-text
